# Microbiota Biomarkers for Lung Cancer

**DOI:** 10.3390/diagnostics11030407

**Published:** 2021-02-27

**Authors:** Qixin Leng, Van K. Holden, Janaki Deepak, Nevins W. Todd, Feng Jiang

**Affiliations:** 1 Department of Pathology, School of Medicine, University of Maryland, 10 S. Pine St., Baltimore, MD 21201, USA; QLeng@som.umaryland.edu; 2 Department of Medicine, School of Medicine, University of Maryland, Baltimore, MD 21201, USA; VHolden@som.umaryland.edu (V.K.H.); jdeepak@som.umaryland.edu (J.D.); ntodd@som.umaryland.edu (N.W.T.)

**Keywords:** microbiome, bacteria, biomarkers, lung cancer, sputum

## Abstract

Non-small cell lung cancer (NSCLC) is the number one cancer killer and its early detection can reduce mortality. Accumulating evidences suggest an etiopathogenic role of microorganisms in lung tumorigenesis. Certain bacteria are found to be associated with NSCLC. Herein we evaluated the potential use of microbiome as biomarkers for the early detection of NSCLC. We used droplet digital PCR to analyze 25 NSCLC-associated bacterial genera in 31 lung tumor and the paired noncancerous lung tissues and sputum of 17 NSCLC patients and ten cancer-free smokers. Of the bacterial genera, four had altered abundances in lung tumor tissues, while five were aberrantly abundant in sputum of NSCLC patients compared with their normal counterparts (all *p* < 0.05). Acidovorax and Veillonella were further developed as a panel of sputum biomarkers that could diagnose lung squamous cell carcinoma (SCC) with 80% sensitivity and 89% specificity. The use of Capnocytophaga as a sputum biomarker identified lung adenocarcinoma (AC) with 72% sensitivity and 85% specificity. The use of Acidovorax as a sputum biomarker had 63% sensitivity and 96% specificity for distinguishing between SCC and AC, the two major types of NSCLC. The sputum biomarkers were further validated for the diagnostic values in a different cohort of 69 NSCLC cases and 79 cancer-free controls. Sputum microbiome might provide noninvasive biomarkers for the early detection and classification of NSCLC.

## 1. Introduction

Lung cancer is the leading cause of cancer-related deaths in men and women [1]. Over 85% lung cancers are non-small cell lung cancer (NSCLC), which mainly consists of squamous cell carcinoma (SCC) and adenocarcinoma (AC). The early detection of NSCLC by low-dose CT (LDCT) followed by the effective treatments can reduce mortality [1]. However, many positive LDCT scans are false alarms and result in multiple examinations and invasive biopsies that carry their own morbidity [1]. The development of noninvasive biomarkers that can accurately diagnose early stage lung cancer remains clinically imperative.

Microbiota is the group of a wide-ranging array of microorganisms, including bacteria, archaea, fungi, and viruses that inhabit various body sites [2]. The microbiome, defined as the collection of microbiota and their genes, plays an important role in health and disease [2]. Microbial agents could cause approximately 20% of the overall cancer burden [2]. For example, infection of Human Papilloma virus, Epstein–Barr virus, *Helicobacter pylori* (*H. pylori*), Escherichia coli and *Fusobacterium nucleatum* lead to a variety of malignancies [2]. In the respiratory tract, there are more than 500 different species of bacteria [3]. Changes of the airway microbiome are attributed to lung tumorigenesis through different mechanisms, such as damage of the local immune barrier, production of bacterial toxins that alter host genome stability, and release of cancer-promoting microbial metabolites [4]. Furthermore, intratumoral microbes may directly affect the growth and metastatic spread of tumor cells [2]. Lung cancer patients have lower microbial diversity and altered abundances of particular bacteria compared with cancer-free individuals [5]. 16S rRNA gene sequencing-based studies [6,7,8,9,10,11,12,13] have identified a set of genera with either higher or lower abundances in lung tumors vs. normal lung tissues. For example, Acidovorax was especially abundant in patients with TP53 mutation-positive lung SCC specimens [8]. The abundances of *Streptococcus* and *Veillonella* were associated with upregulation of the ERK and PI3K signaling pathways in NSCLC cells [12]. In addition, saliva of lung cancer patients possessed an elevated abundance of *Capnocytophaga*, *Selenomonas*, *Veillonella*, *Sphingomonas*, and *Blastomonas* [14,15]. Moreover, *Granulicatella*, *Abiotrophia*, and *Streptococcus*, *Adiacens*, *Intermedius*, and *Mycobacterium tuberculosis* were enriched in the sputum of lung cancer patients [16,17,18]. Importantly, since the microbiota of an individual is stable long-term, they might provide biomarkers for lung cancer [2,3,11,17,19,20].

Two major types of specimens, lower (e.g., bronchoalveolar lavage (BAL and lung tumor tissues)) and upper (saliva and buccal samples) respiratory tract samples, are used for the development of lung cancer biomarkers. BAL and lung tumor tissues are invasively collected via a bronchoscopy or surgery, and thus are not suitable for the development of noninvasive biomarkers. Saliva and buccal samples are obtained less invasively. However, when used for diagnosis of lung cancer, the microbiome in the specimens will be confounded by oral contamination. In contrast, sputum is readily available and can be self-collected. Furthermore, since sputum is secreted from bronchi and bronchioles of the lower respiratory tract, it is more objective and representative than saliva and buccal samples in reflecting the microbial environment of the lungs. Thus, sputum is a viable option for sampling of the lung microbiome without oral contamination. In addition, since molecular changes detected in sputum could reflect those in low respiratory tract [21,22,23,24,25,26,27,28,29,30,31,32,33,34,35,36,37,38,39,40], sputum can be substituted for the lower-airway fluids (e.g., BAL and surgical tissues), which are more invasively collected, for sensitive detection of lung cancer. Therefore, sputum has the advantages as surrogate material and overcomes the obstacles of the commonly used specimens for diagnosis of lung cancer. Taking advantage of the lung cancer-associated genera identified by previous studies [3,5,6,7,8,10,11,12,13,14,15,17,19,41,42], herein we aimed to evaluate the potential of microbiome as sputum biomarkers for lung cancer.

## 2. Materials and Methods

### 2.1. Study Population

The study protocol was approved by the Institutional Review Board of the University of Maryland Medical Center (IRB HP-00040666). From a tissue bank, we obtained 31 frozen lung tumor and the matched noncancerous lung tissues of stage I NSCLC patients who had either a lobectomy or a pneumonectomy. Tumor tissues were intraoperatively dissected from the surrounding lung parenchyma. Paired normal lung tissues were obtained from the same patients at an area distant from their tumors. Of the 31 cases, 16 cases were diagnosed with SCC and 15 were AC of the lungs. We collected sputum samples from participants between the ages of 55–79 at the point of their referral for suspected lung cancer. A total of 27 subjects including 17 lung cancer patients and ten cancer-free smokers were recruited. The 17 lung cancer patients were diagnosed with NSCLC consisting of five stage I cases, five stage II cases, and seven stage III-IV cases. The NSCLC cases consisted of ten AC and seven SCC of lungs (Table 1). The ten cancer-free patients were smokers who had either granulomatous inflammation (*n* = 5), nonspecific inflammatory changes (*n* = 3) or pulmonary infections (*n* = 2).

Sputum samples of 69 lung cancer patients and 79 cancer-free smokers were obtained from Dr. Ruth L Katz’s laboratory of The University of Texas M.D. Anderson Cancer Center. As shown in Table 2, the 69 NSCLC patients consisted of 22 stage I cases, 24 stage II cases, and 23 stage III–IV cases. Thirty-six cases were AC and 33 were SCC of the lungs. The 79 cancer-free patients who were smokers and had either granulomatous inflammation (*n* = 39), nonspecific inflammatory changes (*n* = 22) or lung infections (*n* = 18).

### 2.2. Collection and Preparation of Sputum

Sputum was collected from the participants before they received any treatment as described in our previous studies [29,30,31,32,33,34,35,36,37,38,43,44,45]. To reduce the percentage of oral epithelial cells in sputum, the participants were asked to blow their nose, rinse their mouth, and swallow water to minimize contamination of squamous cells from postnasal drip and saliva. Sputum samples were then coughed into a sterile container and processed within 2 h. To further minimize oral squamous cell contamination, opaque or dense portions that looked different from saliva under the inverted microscope were selected using blunt forceps from expectorate. The samples were processed on ice in four volumes of 0.1% dithiothreitol (Sigma-Aldrich, St. Louis, Mo) followed by four volumes of phosphate-buffered saline (Sigma-Aldrich). We centrifuged the samples at 1500× *g* for 15 min and removed the supernatant. The remaining cell pellets were collected and stored at −80 °C until use.

### 2.3. Genomic DNA Isolation

We used QIAGEN-DNeasy Blood & Tissue Kit (QIAGEN, Germantown, MD, USA) to isolate DNA from the cell pellets or tissue specimens according to manufacturer’s instructions [23,46,47]. We determined the purity by taking the optical density (OD) of the sample at 280 nm for protein concentration and at 260 nm for DNA concentration. The ratio OD260 /OD280 was calculated and DNA sample within the range of 1.6–2 was considered as pure.

### 2.4. Detection and Quantification of Bacterial Abundances Using Droplet Digital PCR (Ddpcr)

We preformed ddPCR to detect DNA of 25 bacterial genera (Table 3) by using a QX100 Droplet Digital PCR System and 2× ddPCR Supermix (Bio-Rad, California, CA, USA) with a protocol developed in our previous studies with modification [23,30,40,46,48,49,50,51,52]. The 25 bacterial genera were suggested to be associated with lung cancer by previous studies (references in Table 3) and thus tested in this study. To design genus-specific primers of PCR test for determining their bacterial abundances, we first aligned 16S rRNA sequences for the maximum number of species for the specific genus to identify consensus regions at genus level. We then use the Primer3 primer design program to design specific primers as previous described [53,54]. Sequences of PCR primers to amplify DNA of the bacterial genera are shown in Table 3. To generate the droplets, we inserted 20 µL of PCR reaction and 70 µL of Droplet Generation oil for Probes (Bio-Rad) in an eight-well cartridge using a QX100 droplet generator (Bio-Rad). We then transferred 40 µL of the generated droplet emulsion in a 96-well PCR plate (Eppendorf, Hamburg, Germany). Amplification reaction was conducted in a T100™ thermal cycler (Bio-Rad) with the following conditions: initial denaturation at 95 °C for 5 min followed by 35 cycles of 15 s at 95.0 °C, 30 s at 55.3 °C, 5 min at 4 °C, and, finally, 5 min at 90 °C for signal stabilization. After thermal cycling, we transferred plates to a droplet reader (Bio-Rad). We used the software provided with the ddPCR system for data acquisition to calculate the concentration of target DNA in copies/µL from the fraction of positive reactions using Poisson distribution analyses.

### 2.5. Statistical Analysis

We used statistical system software version 6.12 (SAS Institute, Cary, NC) and GraphPad Prism version 7 (GraphPad Software, La Jolla, CA) for data analysis. The results were graphed and plotted by GraphPad Prism version 7. Mann–Whitney U test was used to determine whether bacterial abundances were significantly different between lung cancer patients and healthy controls. Furthermore, Pearson’s correlation coefficient test was used to determine the associations of bacterial abundances with clinicopathologic and demographic characteristics of the participants. Spearman correlation test was carried out to analyze the correlation between abundances of bacterial genera. Logistic regression was used to generate prediction models. To evaluate diagnostic significance of potential biomarkers, we used receiver-operator characteristic (ROC) curve analysis and computed the area under ROC (AUC) value by numerical integration of the ROC curve.

## 3. Results 

### 3.1. Bacterial Genera Displayed Different Abundances between Lung Tumor and Noncancerous Lung Tissues

We have proven that ddPCR is a direct method for absolutely and quantitatively measuring nuclear acids without requiring internal control genes and calculating standard curves, which simplifies experimentation and data comparability [23,30,40,46,48,51]. Furthermore, ddPCR had a higher sensitivity compared with conventional PCR for detection and quantification of nuclear acids [23,30,40,46,48,51]. Therefore, in this study, we used ddPCR to determine DNA abundances of 25 bacterial genera (Table 2), whose changes were suggested to be associated with lung cancer [3,5,6,7,8,10,11,12,13,14,15,17,19,41,42]. All the bacterial genera tested by ddPCR generated at least 10,000 droplets in each well of reaction and, therefore, were successfully “read” by ddPCR for the absolute quantification in the tissue specimens. 

**Figure 1:**
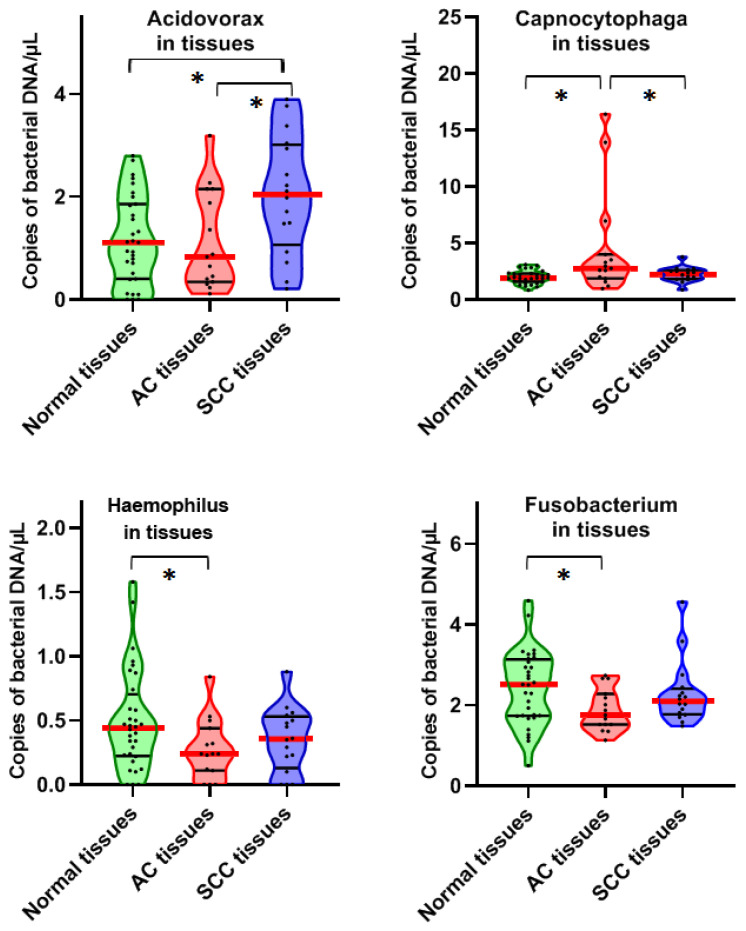
Four bacterial genera show different abundances between lung AC and SSC tissues and the matched normal lung tissues. Solid red line indicates median, while black line indicates quartiles of abundance (copies of bacterial DNA/µL) of each genera in the different types of specimens. * shows that the *p*-value is under 0.05 by a Mann–Whitney U test.

As shown in Figure 1, *Acidovorax* was overrepresented in SCC tissues compared with noncancerous lung tissues and AC tissues (*p* = 0.0051). Capnocytophaga DNA was enriched in AC tissues compared with noncancerous lung tissues and SCC tissues (*p =* 0.0049) (Figure 1). However, the abundances of *Haemophilus* and *Fusobacterium* were lower in AC tissues compared with noncancerous lung tissues and SCC tissues (*p =* 0.049 and 0.039), respectively (Figure 1).

### 3.2. Bacterial Genera Displayed Different Abundances in Sputum of Lung Cancer Patients vs. Cancer-Free Smokers

**Figure 2:**
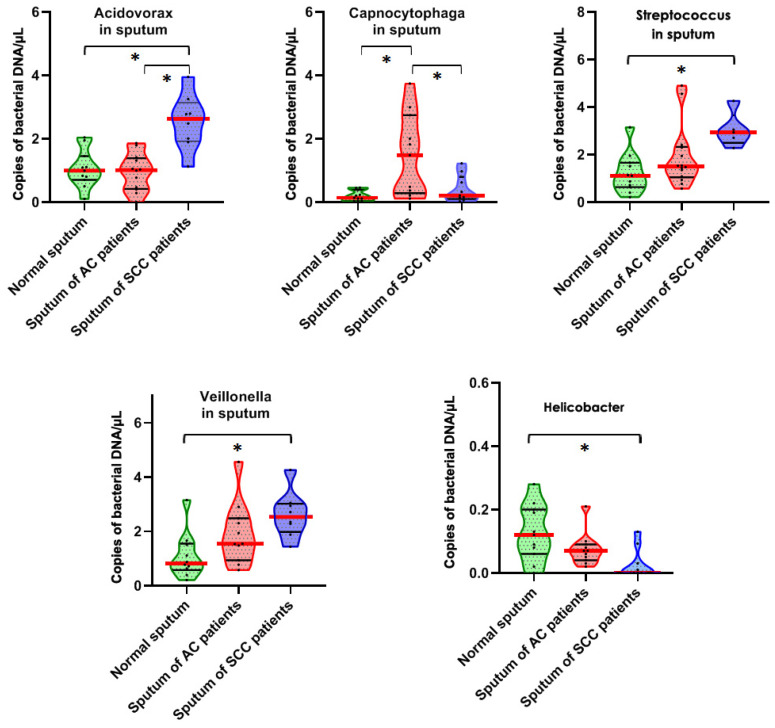
Five bacterial genera show different abundances in sputum of lung AS or SCC patients and cancer-free individuals. The solid red line indicates median, while the black line indicates quartiles of abundance (copies of bacterial DNA/µL) of each genera in the specimens. *, *p* < 0.05 determined by a Mann–Whitney U test).

All the 25 bacteria produced more than 10,000 droplets in each reaction, and thus were also readily detected in the sputum specimens by ddPCR. Of the bacteria, *Acidovorax*, *Streptococus*, and *Veillonella* were overrepresented in sputum of lung SCC patients compared with lung AC patients and cancer-free smokers (pall *p* < 0.05) (Figure 2). The abundance of *Helicobacter* was underrepresented in sputum of lung SCC patients compared with lung AC patients and cancer-free smokers (*p=* 0.018 (Figure 2). *Capnocytophaga* was enriched in sputum of lung AC patients compared with lung SCC patients and cancer-free smokers (*p =* 0.046) (Figure 2).

Furthermore, both *Acidovorax* and *Capnocytophaga* displayed significantly different abundances in sputum of lung AC vs. SCC patients (all *p* < 0.05). In addition, abundances of the five sputum bacteria were not associated with the age, gender, ethnic group, tumor stage, and smoking status of the patients (all *p* > 0.05), except histology and location of primary lung tumors (all *p* > 0.05).

Comparison of abundances of bacteria in tumor tissues of lung cancer patients and sputum of lung cancer patients and cancer-free smokers.

**Figure 3:**
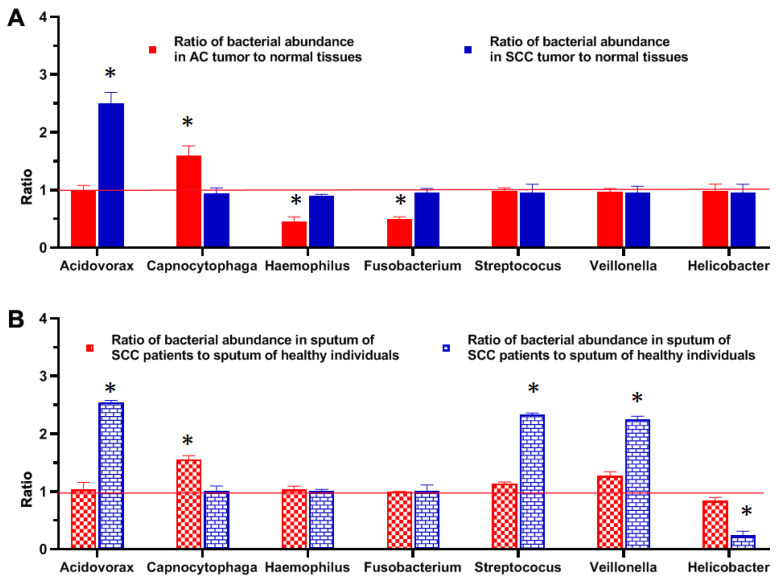
Comparison of abundances of bacteria in tumor tissues of lung cancer patients and sputum of lung cancer patients and controls. Relative abundances of bacterial genera in tumor tissues (**A**) and sputum of lung cancer patients (**B**) as compared with their normal counterparts. High abundances of bacterial genera in tumor tissue or sputum specimens are shown above ratio of 1 (red line), whereas low abundances in tumor tissue or sputum specimens are shown below ratio of 11 (red line). *, *p* < 0.05 determined by a Mann–Whitney U test).

The change of *Acidovorax* abundance had a similar trend in SCC tissues as in sputum of lung SCC patients (Figure 3) (Spearman correlation test, *p =* 0.023). Furthermore, *Capnocytophaga* had a similar trend in AC tissues as in sputum of lung AC patients. (Spearman correlation test, *p =* 0.017). The altered abundances of the two bacterial genera (*Acidovorax* and *Capnocytophaga*) in sputum might directly reflect those in lung tumor tissues. However, the reduced abundances of *Haemophilus* and *Fusobacterium* were only observed in lung AC tissue specimens compared with their normal counterparts (Figure 3A). The increased abundances of the *Streptococcus* and *Veillonella* were solely discovered in sputum of lung SCC patients and decreased abundances of *Helicobacter* were found in sputum of lung AC patients, as compared with their normal counterparts (Figure 3B).

### 3.3. Development of Sputum Bacteria Biomarkers for NSCLC

Sputum is noninvasively obtained body fluid. It contains bronchial epithelial cells from the lungs and lower respiratory tract and, thus, has the advantages as surrogate material for specifically diagnosing lung cancer. We evaluated if the five bacteria, which were readily detected in sputum and associated with lung cancer, could be used as noninvasive biomarkers for NSCLC. In the cohort 1 of sputum specimens, the five bacteria exhibited AUC values of 0.56–0.88 in distinguishing NSCLC patients from controls (Table 4).

**Figure 4:**
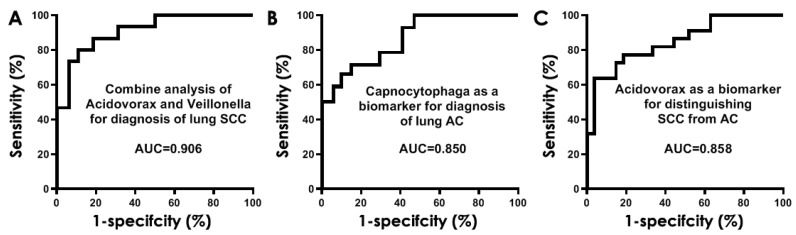
Receiver-operator characteristic (ROC) curve analysis of abundances of bacterial genera in sputum of cohort 1 comprising NSCLC patients and cancer-free controls. The area under the ROC curve (AUC) for bacterial genera conveys its accuracy for diagnosis and classification of NSCLC. (**A**) The combined use of *Acidovorax* and *Veillonella* produced 0.91 AUC. (**B**) The use of *Capnocytophaga* as a sputum biomarker could detect lung AC with 0.85 AUC. (**C**) The use of *Acidovorax* as a sputum biomarker had 0.85 AUC for differentiating between SCC and SC of the lungs.

We used a stepwise logistic regression analysis to select the optimal panels of biomarkers. Two bacteria consisting of *Acidovorax* and *Veillonella* were selected as the best biomarkers for lung SCC. The two bacterial biomarkers used in combination produced 0.91 AUC (Figure 4A) in diagnosis of lung SCC with 80.00% sensitivity and 89.26% specificity (Table 4). The estimated correlations among levels of the two bacteria were very low (Spearman correlation test, *p =* 0.53), implying that the integration of the two biomarkers has complementary classification. Furthermore, the use of *Capnocytophaga* as a sputum biomarker could detect lung AC with 0.85 AUC (Figure 4B), 72.73% sensitivity and 85.19% specificity (Table 4).

In addition, the use of *Acidovorax* as a sputum biomarker had 0.86 AUC (Figure 4C) with 63.64% sensitivity and 96.30% specificity for distinguishing between SCC and AC of the lungs (Table 5). The bacterial biomarkers had no association with age, gender, and smoking status of the participants, and stages of lung tumors (Pearson’s correlation coefficient test, all *p* > 0.05), except location and histology of primary lung tumors (Supplementary Table S1).

### 3.4. Validating the Bacterial Biomarkers in an Independent Set of Lung Cancer Patients and Controls

The sputum bacterial biomarkers developed from the cohort 1 were tested using the same procedures to diagnose lung cancer in cohort 2 consisting of 69 NSCLC patients and 79 controls. Consistent with findings in the cohort 1, abundances of *Acidovorax*, *Streptococus*, and *Veillonella* were higher in sputum of lung SCC patients compared with lung AC patients and cancer-free smokers (all *p* < 0.05). The abundance of *Helicobacter* was lower in sputum of lung SCC patients compared with lung AC patients and cancer-free smokers (*p =* 0.018). *Capnocytophaga* was overrepresented in sputum of lung AC patients compared with lung SCC patients and cancer-free smokers (*p =* 0.046).

**Figure 5:**
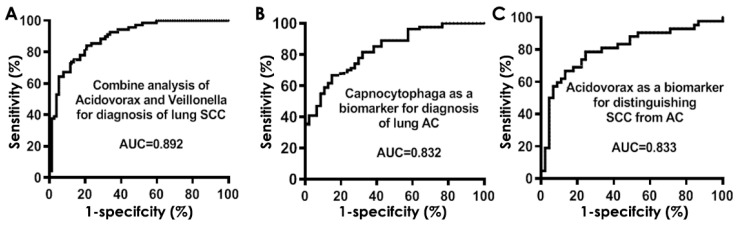
ROC curve analysis of abundances of bacterial genera in sputum of cohort 2. (**A**) The combined use of *Acidovorax* and *Veillonella* produced 0.89 AUC. (**B**) The use of *Capnocytophaga* as a sputum biomarker could detect lung AC with 0.83 AUC. (**C**) The use of *Acidovorax* as a sputum biomarker had 0.83 AUC for differentiating between SCC and SC of the lungs.

Furthermore, the bacterial biomarkers displayed similar diagnostic values in the cohort 2 as did in the cohort 1 (Figure 5). Particularly, *Acidovorax* and *Veillonella* used in combination could diagnose lung SCC with 0.89 AUC, producing 75.76% sensitivity and 88.61% specificity (Table 5). In addition, sputum *Capnocytophaga* biomarker could detect lung AC with 0.83 AUC, yielding 69.44% sensitivity and 84.42% specificity (Table 5). Moreover, the use of *Acidovorax* as a sputum biomarker had 0.83 AUC with 66.67% sensitivity and 89.86% specificity for distinguishing between SCC and AC of the lungs. There was no association of sputum bacterial genera with age, gender, and smoking status of the participants, and stages of lung tumors (all *p* > 0.05), except location and histology of primary lung tumors (Supplementary Table S2). There was no statistical difference of sensitivity and specificity of combined use of *Acidovorax* and *Veillonella* for diagnosis of SCC and using *Capnocytophaga* for detection of AC (all *p* > 0.05) in the cohort 1 and cohort 2. There was also no statistical difference of sensitivity and specificity of using *Capnocytophaga* for detection of AC (all *p* > 0.05) in the cohort 1 and cohort 2. However, the use of sputum Acidovorax had a lower specificity in cohort 2 compared with cohort 1 for distinguishing between SCC and AC of the lungs (89% vs. 96%, *p =* 0.02), while maintaining a similar sensitivity (63% vs. 66%) (Table 5). 

## 4. Discussion 

Our present study confirms that certain microbes, at genus level, are differentially abundant in lung tumor vs. normal lung tissues. Furthermore, we demonstrate the abundances of genera could be quantitatively measured in sputum by using ddPCR and the altered abundances of some sputum bacteria are associated with lung cancer. We further develop *Acidovorax* and *Veillonella* as a sputum biomarker panel for lung SCC, regardless of the stages. In addition, a single sputum bacterial biomarker, *Capnocytophaga*, could be used for detection of lung AC. Moreover, the use of *Acidovorax* as a sputum biomarker could distinguish between SCC and AC, the two major histological types of NSCLC. Therefore, the sputum microbiota might have the potential use as noninvasive biomarkers for diagnosis and classification of lung cancer at the early stage. 

Previous studies have shown that diverse airway microbial profiles exist at different airway sites of lung cancer patients [42,68,69]. However, the comparison of bacterial profiles in primary tumor tissues and sputum of lung cancer patients has not been performed. Our findings in comparison of bacterial abundances in tumor tissues and sputum of lung cancer patients suggest that the altered bacterial genera could be classified into three categories. (1) Lung tumor microbes, which comprise *Capnocytophaga* and *Haemophile*. Aberrant abundances of the bacterial genera were exclusively found within lung tumors. The intratumoral microbes of lung cancer might be directly involved in the development and progressions of NSCLC, however, the imbalance in their abundance is not detectable in sputum. (2) Sputum microbes of lung cancer patients, such as *Streptococcus*, *Veillonella*, and *H. pylori*, whose aberrations were solely observed in sputum of NSCLC patients. Changes of these microbes in sputum might not simply mirror those in primary lung tumors. The discovery is in line with the previous observation [70]. The analysis of bladder tumors and the paired urine samples showed that aberrations of certain bacteria existed in urine rather than the tumor tissues, however, they had diagnostic significance for malignancy [70]. This category of microbiota might indirectly prompt tumor susceptibility and development via altering respiratory bacterial environment and modulating inflammation, inducing DNA damage, and producing metabolites involved in oncogenesis or tumor suppression [2]. (3) Bacterial genera whose changes in sputum were consistent with those in tumor tissues in the same direction, including *Acidovorax* and *Capnocytophaga*. The aberrant bacterial abundances in sputum could directly reflect those in primary lung tumors. We have also found that altered abundances of the bacterial genera in sputum are histologically dependent. Particularly, the abundances of sputum *Acidovorax*, *Streptococcus*, *H. pylori*, and *Veillonella* in sputum are related with lung SCC, whereas increased *Capnocytophaga* abundance in sputum is related to lung AC. Nevertheless, an extensive and deep investigation of the microbiota is needed to have a better understanding of the pathogenesis of NSCLC and provide new diagnostic and therapeutic targets for the disease.

Overall, the potential sputum bacterial biomarkers have a higher sensitivity for lung SCC compared with lung AC (80.00% vs. 72.73% *p =* 0.032). The findings are in good agreement with our previous studies [6,23,27,30,32,34,40,46,50,52]. We have shown that sputum-based molecular biomarkers have a higher sensitivity in identifying central SCC compared with peripheral AC of the lungs. The possible reason might be that sputum is secreted from large airways or main bronchi where SCC more commonly exists. Conversely, lung AC tumors often arise in peripheral lung tissue and originate from the smaller airways of the lungs. Future integration of the sputum-based assay with LDCT could overcome the weakness of the imaging analysis by improving accuracy for the early detection of lung SCC. 

Among the bacterial genera analyzed, Acidovorax was found by Greathouse et al. to have an elevated abundance in lung SCC tissues with TP53 mutation [8]. Furthermore, there was a significant increase in lung tumor volume in mice inoculated with *Acidovorax* temperans. *Acidovorax* temperans could contribute to lung carcinogenesis in the presence of activated Kras and mutant p53 and, thus, act as a promoter in the development and progression of the disease [8]. Our current study supports this early report [8], and more importantly, suggests that *Acidovorax* might provide a sputum biomarker for lung SCC. *Capnocytophaga* species were proposed to be involved in lung carcinogenesis and lower respiratory tract infections [15]. Furthermore, *Capnocytophaga* might induce long-term immune response/infection to the organ or cancer growth environment, which favors the growth of these bacteria in the airways [68]. Our study also suggests that *Capnocytophaga* abundance is significantly higher in NSCLC vs. normal lung tissues. Tsay et al. found an increased abundance of *Streptococcus* and *Veillonella* in the lower respiratory tract of NSCLC patients, which was associated with upregulation of the ERK and PI3K signaling pathways [12]. It has been well accepted that *H. pylori* is a risk factor for gastric and several other cancers [2]. Our present study demonstrates a close association of *H. pylori* with lung SCC. However, rigorous investigations regarding the *H. pylori*-lung cancer association remain to be performed. 

Smoking causes most lung cancers, but lung cancer can be found in never smokers [1]. Interestingly, the abundances of the genera tested in this present study were not associated with the smoking status of the patients. The result suggests that the microbiota aberrations might play an important role in lung tumorigenesis of nonsmokers. The observation is in line with previous studies [16,17], in which lung cancer patients who were never smokers had a long history of bacterial respiratory tract infection. Dysregulation of the genera could be involved in the development and progression of NSCLC via a specific manner that is beyond tobacco-smoking-related carcinogenesis. 

The sputum biomarkers were further tested in an independent (validation) cohort of cases and controls. The diagnostic significance of the bacterial biomarkers for diagnosis of SCC and AC of the lungs was confirmed. However, although the use of sputum Acidovorax for distinguishing between SCC and AC had a similar sensitivity, its specificity was reduced in the validation cohort. Possible explanation for the difference might be that the sputum specimens of the validation cohort were collected five years ago, whereas sputum samples in cohort 1 were fresh and collected within six months. DNA quality might significantly decline with long storage duration, leading to a lower specificity of the sputum biomarker for distinguishing between SCC and AC of the lungs. 

This study may suffer some limitations. (1), the sample size is small. We will prospectively validate the sputum biomarkers in a large cohort. (2), we only assessed 25 bacterial genera whose changes were previously suggested to be associated with lung cancer. Although the results show promise, the sensitivity and specificity of the biomarkers are not enough in routine laboratory settings. We will evaluate more lung tumor-bacteria to identify additional bacterial biomarkers that can be added to the current ones so that the diagnostic efficacy of the sputum tests could be improved. 

## 5. Conclusions

We show that aberrant microbial composition, at genus level, is present in lung tumor and sputum of lung cancer patients. We have for the first time developed sputum bacterial biomarkers that could be potentially used for the early detection and classification of lung cancer, though a larger sample study is needed to validate the findings.

## Figures and Tables

**Table 1:** Cohort 1 of NSCLC patients and cancer-free smokers from whom sputum specimens were collected.

	NSCLC Cases (*n* = 17)	Controls (*n* = 10)	*p*-Value
Age	66.37 (SD 9.05)	61.27 (SD 9.46)	0.36
Sex			0.43
Female	7	4	
Male	10	6	
Race			0.39
African Americans	5	3	
White American	12	7	
Smoking pack-years (median)	32.17	28.38	0.26
Stage			
Stage I	5		
Stage II	5		
Stage III-VI	7		
Histological type			
Adenocarcinoma	10		
Squamous cell carcinoma	7		
Location of primary lung tumors			
Peripheral location	10		
Central location	7		

Abbreviations: NSCLC, non-small cell lung cancer; Central location: limited to the trachea, bronchi, or segmental bronchi. Peripheral location: limited more to the periphery than the subsegmental bronchi.

**Table 2:** Cohort 2 of NSCLC patients and cancer-free smokers from whom sputum specimens were collected.

	NSCLC Cases (*n* = 69)	Controls (*n* = 79)	*p*-Value
Age	64.18 (SD 6.25)	63.29 (SD 6.24)	0.32
Sex			0.35
Female	26	30	
Male	43	49	
Smoking pack-years (median)	35.25	33.29	0.41
Stage			
Stage I	22		
Stage II	24		
Stage III–VI	23		
Histological type			
Adenocarcinoma	36		
Squamous cell carcinoma	33		
Location of primary lung tumors			
Peripheral location	36		
Central location	33		

Abbreviations: NSCLC, non-small cell lung cancer; Central location: limited to the trachea, bronchi, or segmental bronchi. Peripheral location: limited more to the periphery than the subsegmental bronchi.

**Table 3:** Twenty-five bacterial genera tested by ddPCR and their primers.

Name	Target Region (Accession #)	Forward (5′-3′)	Reward (5′-3′)	References
Acidovorax	NZ_LJGO01000014.1	GTCATCCTCCACCAACCAATAC	GTCTATACCGGACCAACAACAA	[8]
Akkermansia	NZ_AP021898.1	CAGCACGTGAAGGTGGGGAC	CCTTGCGGTTGGCTTCAGAT	[55]
Bacteroides	NZ_VKLY01000054.1	GACCGCATGGTCTTGTTATT	CGTAGGAGTTTGGACCGTGT	[56]
Bifidobacterium	NZ_AKCA01000001.1	CCACATGATCGCATGTGATTG	CCGAAGGCTTGCTCCCAAA	[56]
Bilophila	NZ_KE150238.1	CGTGTGAATAATGCGAGGG	TCTCCGGTACTCAAGCGTG	[57]
Blautia	NZ_NQOF01000001.1	GTGAAGGAAGAAGTATCTCGG	TTGGTAAGGTTCTTCGCGTT	[58]
Bradyrhizobium	NZ_VSSR01000023.1	ATCGACGTGCTGCCAATAA	GCCGATAACAAGACGGAAATAAC	[13]
Capnocytophaga	NZ_BLBC01000010.1	TGGWCAATGGTCGGAAGACTG	CCGCTACACTACACATTCCA	[9]
Curvibacter	NZ_CP022389.1	GAGCCTTTACCTCACCAACTAC	CGTAGCGAAAGCTACGCTAATA	[59]
Enterococcus	NZ_CP023011.2	GGCATATTTATCCAGCACTAG	TAGCGTACGAAAAGGCATCC	[17]
Escherichia	NC_000913.3	CATGCCGCGTGTATGAAGAA	CGGGTAACGTCAATGAGCAAA	[17]
Faecalibacterium	NZ_CP030777.1	GGAGGAAGAAGGTCTTCGG	AATTCCGCCTACCTCTGCACT	[60]
Fusobacterium	NZ_LT608325.1	AAGCGCGTCTAGGTGGTTATGT	TGTAGTTCCGCTTACCTCTCCAG	[61]
Haemophilus	NZ_LS483458.1	AGCGGCTTGTAGTTCCTCTAACA	CAACAGAGTATCCGCCAAAAGTT	[62]
Helicobacter	NC_017379.1	GCGCATGTCTTCGGTTAAAAA	TTCCATAGGCTATAATGTGATCCAAA	[63]
Klebsiella	NZ_CP023478.1	CGGGCGTAGCGCGTAA	GATACCCGCATTCACATTAAACAG	[64]
Lactobacillus	NZ_MWIK01000038.1	CGCCACTGGTGTTCYTCCATATA	AGCAGTAGGGAATCTTCCA	[65]
Mycobacterium	NZ_UATA01000019.1	CAAGCGGTGGAGCATGTG	CTAAGATGTCAAACGCTGGTAAGG	[66]
Neisseria	NZ_UGRT01000005.1	CTGTTGGGCARCWTGAYTGC	GATCGGTTTTRTGAGATTGG	[7]
Prevotella	NZ_BAKG01000039.1	CCTACGATGGATAGGGGTT	CACGCTACTTGGCTGGTTCAG	[5]
Pseudomonas	NZ_BMDE01000022.1	CAGCCATGCCGCGTGTGTGA	GTTGGTAACGTCAAAACAGCAAGG	[67]
Ruminococcus	NZ_QRIH01000002.1	GCTTAGATTCTTCGGATGAAGAGGA	AGTTTTTACCCCCGCACCA	[68]
Selenomonas	NZ_JH376859.1	ACRCGTAGRCAACCTGCCG	CGATCCGAAGACCTTCTTCAC	[15]
Streptococcus	NZ_UYIP01000002.1	ACGCAATCTAGCAGATGAAGCA	TCGTGCGTTTTAATTCCAGC	[7,16]
Veillonella	NZ_AUAN01000022.1	CGGGTGAGTAACGCGTAATCA	CCAACTAGCTGATGGGACGC	[15]

**Table 4:** The five bacterial genera display different levels in sputum samples of NSCLC patients vs. cancer-free controls of cohort 1.

Genera	*p* Value of Ac Patients vs. Controls	*p* Value of Scc Patients vs. Controls	AUC of AC Patients vs. Controls	AUC of SCC Patients vs. Controls
*Acidovorax*	0.7090	0.0015	0.5636	0.8814
*Capnocytophaga*	0.0455	0.3194	0.8502	0.6833
*Helicobacter*	0.0705	0.0175	0.7273	0.8070
*Streptococcus*	0.2775	0.0042	0.6753	0.8117
*Veillonella*	0.1086	0.0098	0.7062	0.8286

Abbreviations: NSCLC, non-small cell lung cancer; AC, adenocarcinoma; SCC, squamous cell carcinoma; AUC, the area under receiver-operator characteristic curve.

**Table 5:** The diagnostic values of the sputum bacterial biomarkers in contorts 1 and 2.

	Cohort 1 of 17 NSCLC Patients and 10 Cancer-Free Controls		Cohort 2 of 69 NSCLC Patients and 79 Cancer-Free Controls	
	Sensitivity	Specificity	Sensitivity	Specificity
Combined Acidovorax and Veillonella for SCC	80.79%	89.08%	75.76%	88.61%
Capnocytophaga for AC	72.70%	85.28%	69.44%	84.42%
Acidovorax for distinguishing SCC from AC	63.64%	96.30%	66.67%	89.86%

## Data Availability

The data that support the findings of this study are available from the corresponding author upon a reasonable request.

## Supplementary Materials

The following are available online at https://www.mdpi.com/2075-4418/11/3/407/s1, Table S1. The association of abundances of bacterial genera in sputum of cohort 1 with the age, gender, ethnic group, tumor stageand location, and smoking status of the patients determined by Pearson’s correlation coefficient test. A *p*-value < 0.05 is statistically significant; Table S2. The association of abundances of bacterial genera in sputum of cohort 2 with the age, gender, ethnic group, tumorstageand location, and smoking status of the patients determined by Pearson’s correlation coefficient test. A *p*-value < 0.05 is statistically significant.
