# Supplementary material for: Microbiota Biomarkers for Lung Cancer
Source: Diagnostics (Basel). 2021 Feb 27;11(3):407. doi: 10.3390/diagnostics11030407 (PMC7997424; doi:10.3390/diagnostics11030407)
Supplement: Supplementary file 1 [file diagnostics-11-00407-s001.pdf]

diagnostics

Microbiota Biomarkers for Lung Cancer

Qixin Leng [1], Van K. Holden [2], Janaki Deepak [2], Nevins W. Todd [2] and Feng Jiang [1,*]

1 Department of Pathology, School of Medicine, University of Maryland, 10 S. Pine St. Baltimore, MD 21201, USA; QLeng@som.umaryland.edu
2 Department of Medicine, School of Medicine, University of Maryland, Baltimore, MD 21201, USA; VHolden@som.umaryland.edu (V.K.H.); jdeepak@som.umaryland.edu (J.D.); ntodd@som.umaryland.edu (N.W.T.)
* Correspondence: fjiang@som.umaryland.edu

Citation: Leng, Q.; Holden, V.K.; Deepak, J.; Todd, N.W.; Jiang, F. Microbiota Biomarkers for Lung Cancer. *Diagnostics* **2021**, *11*, 407. https://doi.org/10.3390/diagnostics11030407

Received: 15 January 2021
Accepted: 23 February 2021
Published: 27 February 2021

Publisher's Note: MDPI stays neutral with regard to jurisdictional claims in published maps and institutional affiliations.

Copyright: © 2021 by the authors. Licensee MDPI, Basel, Switzerland. This article is an open access article distributed under the terms and conditions of the Creative Commons Attribution (CC BY) license (http://creativecommons.org/licenses/by/4.0/).

Table S1. The association of abundances of bacterial genera in sputum of cohort 1 with the age, gender, ethnic group, tumor stage and location, and smoking status of the patients determined by Pearson's correlation coefficient test. A p-value < 0.05 is statistically significant.

	**Age**	**Gender**	**Race**	**Smoking status**	**Stage**	**Histology**	**Primary lung tumor location**
Genera	Correlation coefficients (P-value)	Correlation coefficients (P-value)	Correlation coefficients (P-value)	Correlation coefficients (P-value)	Correlation coefficients (P-value)	Correlation coefficients (P-value)	Correlation coefficients (P-value)
Acidovorax	−1.002 (P=0.311)	−0.199 (P=0.414)	−0.484 (P=0.355)	−0.543 (P=0.359)	−0.592 (P=0.336)	−0.757 (P=0.020)	−0.757 (P=0.020)
Streptococus	−0.807 (P=0.452)	0.269 (P=0.522)	−0.246 (P=0.768)	−0.817 (P=0.614)	−0.427 (P=0.263)	0.532 (P=0.012)	0.532 (P=0.012)
Veillonella	−0.346 (P=0.225)	−0.857 (P=0.296)	0.102 (P=0.275)	−0.508 (P=0.255)	0.876 (P=0.641)	−0.735 (P=0.023)	−0.735 (P=0.023)
Helicobacter	0.472 (P=0.387)	0.184 (P=0.239)	−0.467 (P=0.521)	−0.422 (P=0.718)	−0.454 (P=0.139)	−0.753 (P=0.004)	−0.753 (P=0.004)
Capnocytophaga	−0.282 (P=0.523)	−0.272 (P=0.462)	−0.482 (P=0.573)	0.571 (P=0.317)	−0.342 (P=0.573)	0.379 (P=0.013)	0.379 (P=0.013)

Table S2. The association of abundances of bacterial genera in sputum of cohort 2 with the age, gender, ethnic group, tumor stage and location, and smoking status of the patients determined by Pearson's correlation coefficient test. A p-value < 0.05 is statistically significant.

	**Age**	**Gender**	**Race**	**Smoking status**	**Stage**	**Histology**	**Primary lung tumor location**
Genera	Correlation coefficients (P-value)	Correlation coefficients (P-value)	Correlation coefficients (P-value)	Correlation coefficients (P-value)	Correlation coefficients (P-value)	Correlation coefficients (P-value)	Correlation coefficients (P-value)
Acidovorax	0.034 (P=0.568)	−0.459 (P=0.645)	−0.451 (P=0.753)	−0.434(P=0.978)	−0.456 (P=0.364)	−0.126 (P=0.023)	−0.126 (P=0.023)
Streptococus	0.268 (P=0.531)	−0.217 (P=0.943)	0.273 (P=0.135)	0.847 (P=0.564)	−0.357 (P=0.430)	−0.801 (P=0.009)	−0.801 (P=0.009)
Veillonella	−0.287 (P=0.532)	0.623 (P=0.732)	−0.345 (P=0.435)	−0.688 (P=0.323)	0.219 (P=0.196)	0.789 (P=0.022)	0.789 (P=0.022)
Helicobacter	0.698 (P=0.415)	−0.136 (P=0.453)	0.537 (P=0.578)	0.359 (P=0.954)	−0.649 (P=0.740)	−0.653 (P=0.017)	−0.653 (P=0.017)
Capnocytophaga	−0.578 (P=0.732)	0.742 (P=0.575)	−0.975 (P=0.232)	−0.789 (P=0.634)	0.537 (P=0.643)	0.529 (P=0.028)	0.529 (P=0.028)
